# Alterations in the phosphodiesterase type 5 pathway and oxidative stress correlate with erectile function in spontaneously hypertensive rats

**DOI:** 10.1111/jcmm.16045

**Published:** 2020-10-29

**Authors:** Weixiang He, Jianmin Liu, Daoquan Liu, Jundong Hu, Ye Jiang, Mingzhou Li, Qian Wang, Ping Chen, Guang Zeng, Deqiang Xu, Xinghuan Wang, Michael E. DiSanto, Xinhua Zhang

**Affiliations:** ^1^ Department of Urology Zhongnan Hospital of Wuhan University Wuhan China; ^2^ Department of Urology First People's Hospital of Xiaochang County Hubei China; ^3^ Department of Urology People's Hospital of Qichun County Hubei China; ^4^ Department of Surgery and Biomedical Sciences Cooper Medical School of Rowan University Camden NJ USA

**Keywords:** corpus cavernosum, erectile dysfunction, hypertension, oxidative stress, phosphodiesterase type 5, spontaneously hypertensive rats

## Abstract

To explore how alterations in the phosphodiesterase type 5 (PDE5) signalling pathway and oxidative stress correlate with changes in the expression of relaxation and contraction molecules and erectile dysfunction (ED) in the corpus cavernosum smooth muscle (CCSM) of spontaneously hypertensive rats (SHR). In this study, SHR and Wistar‐Kyoto (WKY) rats were used. Erectile function was determined by apomorphine test and electrical stimulation (ES) of cavernous nerve. Masson's trichrome staining and confocal microscopy were performed. Nitric oxide synthase (NOS), PDE5, phosphorylated‐PDE5 and α1‐adrenergic receptor (α1AR) were determined by RT‐PCR and Western blotting while oxidative stress in CC was determined by colorimetric analysis. SHR exhibited obvious ED. CC of SHR showed less SM but more collagen fibres. The expression of NOS isoforms in SHR was significantly decreased while all α1AR isoforms were increased. In addition, PDE5 and phosphorylated‐PDE5 were down‐regulated and its activity attenuated in the hypertensive rats. Meanwhile, the SHR group suffered oxidative stress, which may be modulated by endoplasmic reticulum stress and NADPH oxidase up‐regulation. Dysregulation of NOS and α1AR, histological changes and oxidative stress in CC may be associated with the pathophysiology of hypertension‐induced ED. In addition, PDE5 down‐regulation may lead to the decreased efficacy of PDE5 inhibitors in some hypertensive ED patients and treatment of oxidative stress could be used as a new therapeutic target for this type of ED.

## 1 INTRODUCTION

Penile erection is a complex psycho‐physiological process involving a series of neural and vascular activities in which contraction and relaxation of corpus cavernosum smooth muscle (CCSM) play an important role. It is well known that the nitric oxide/cyclic guanosine monophosphate (NO/cGMP) pathway predominantly modulates CCSM relaxation and penile erection.^1^ Specifically, NO is produced by nitric oxide synthase (NOS) using l‐arginine and oxygen. NO increases the production of cGMP (the second messenger), which relaxes the CCSM.^2^ The cGMP is degraded by phosphodiesterase type 5 (PDE5). Currently, the most effective drugs for treating erectile dysfunction (ED) are PDE5 inhibitors (PDE5is). PDE5is can block the activity of the PDE5 enzyme to increase the cGMP level, leading to the relaxation of CCSM and erection. In corpus cavernosum (CC), NOS exists as three isoforms: eNOS (endothelial NOS), nNOS (neuronal NOS) and iNOS (inducible NOS). The nNOS isoform is preferentially expressed in neurons or nerves and considered to mediate both the initiation and maintenance of penile erection, while the eNOS isoform is mainly expressed in endothelial cells and considered responsible for the maintenance of erection.^3^, ^4^, ^5^ iNOS is expressed in almost all cell types. Although recent evidence suggested that iNOS in the penis exhibits an antifibrotic role and improves erectile function in diabetes mellitus‐induced ED,^6^, ^7^ the functions of iNOS in CC were not fully explored. On the other hand, the CCSM spends the majority of its time in the contracted state, which is in contrast with other smooth muscles.^8^ Although the relaxation of CCSM has been extensively studied throughout the last couple of decades, the contraction of CCSM is also important but less studied. Many molecules and pathways are involved in the process of CCSM contraction with adrenergic neurotransmission the most important,^9^ including three major isoforms of α1‐receptors (1a, 1b and 1d) identified in CC.^10^ In general, changes in the expression and function of molecules in the relaxation and contraction pathways may cause an imbalance between the contraction and relaxation of CCSM, eventually resulting in ED.

The risk factors for ED include ageing, psychiatric/psychological disorders, smoking, medications, hormonal factors and some chronic diseases like diabetes mellitus and hypertension.^11^, ^12^ It is well‐known that hypertension is an important worldwide health problem which is more common in elderly people and it can be an independent risk factor for ED.^13^, ^14^ CC is a part of the vascular system therefore ED is closely related to cardiovascular diseases such as hypertension. Indeed, the high prevalence of ED in hypertensive patients was well defined in previous study.^15^ Interestingly, ED is an early marker of hypertension and other cardiovascular diseases.^16^, ^17^ Although ED and hypertension share many similar underlying pathological mechanisms, including endothelial dysfunction, inflammation and atherosclerosis,^17^, ^18^ the mechanism of ED induced by hypertension remains controversial.

Hypertension can impair normal erectile function from both a ‘functional’ and a ‘structural’ aspect. In the hypertensive state, blood vessels often undergo remodelling, including in the penis which is also a vascular organ. In the hypertensive rat, studies showed that the penis exhibited morphological changes and tissue remodelling.^19^, ^20^, ^21^ With regard to functional activity, a number of vasodilators and vasoconstrictors, such as NO, hydrogen sulphide (H_2_S), angiotensin II (AngII), endothelin‐1 (ET‐1), were dysregulated or dysfunctional in CC.^22^, ^23^ As the strongest vasoconstrictor, ET‐1 may also constrict the internal pudendal artery (the major supplying blood vessel of the penis) and reduce blood flow to penis.^24^ Moreover, the activation of the RhoA/ROCK pathway was found to contribute to hypertensive ED.^25^

In recent years, more studies demonstrated that oxidative stress related to hypertension may act as a pathophysiological insult.^26^ The occurrence of oxidative stress is due to an imbalance between the reactive oxygen species (ROS) level and antioxidant activity. ROS includes superoxide, hydrogen peroxide and others. Nicotinamide adenine dinucleotide phosphate (NADPH) oxidase (Nox) is reported to be a major source of ROS in the vascular wall.^27^ The excessive ROS will be cleared by antioxidants in the body, such as superoxide dismutase (SOD) and catalase. Otherwise, they will have adverse effects on cells. Recent evidence showed that Nox subunits were up‐regulated in vascular smooth muscle cells (VSMC) cultured from the spontaneously hypertensive rat (SHR), which is involved in endoplasmic reticulum (ER) stress.^28^, ^29^ However, the role of oxidative stress in CC dysfunction remains unknown.

Currently, the most commonly used PDE5is include sildenafil, vardenafil and tadalafil, which has less side effect and can be delivered on‐demand. Although PDE5is are the first‐line treatment for ED patients, almost 35% of patients show low or no response to PDE5is,^30^ which has perplexed clinicians and patients alike. Recent studies also revealed that PDE5is have less of an effect in hypertensive patients, especially in patients older than 65 years old.^31^, ^32^ Actually, responsiveness to PDE5is is dependent on NO/cGMP pathway integrity and PDE5 enzyme expression. Our previous studies reported that castrated rats^33^ and diabetes mellitus rats^34^ exhibited hypo‐responsiveness to PDE5is because PDE5 protein was down‐regulated in those rat models. Hence, it will be intriguing to determine the activity of PDE5 in the hypertensive CC.

Thus, the mechanism of ED induced by hypertension remains controversial. Our current study aims to explore the effects of hypertension on CC with emphasis on the NO/cGMP/PDE5 axis and oxidative stress.

## 2 MATERIALS AND METHODS

### 2.1 Experimental animal

In total, thirty 12‐week‐old male spontaneously hypertensive rats (SHR) were used as a hypertension rat model and 30 age‐matched male Wistar‐Kyoto rats (WKY) were used as a normotensive control. All animals were specific‐pathogen‐free (SPF) grade. After purchase from Beijing Vital River Laboratory Animal Technology Co., Ltd. (Beijing, China), all animals were kept fed normal chow with a 12 hours day/night light cycle for more than one week to adapt to a new environment. Animal experiments were conducted at the Animal Center of Zhongnan Hospital of Wuhan University and all animal protocols were approved by the Medical Ethics Committee for Experimental animals of Zhongnan Hospital of Wuhan University. The suffering of experimental rats was kept to a minimum.

### 2.2 Apomorphine (APO) test

At the end of 12 weeks, 10 SHR and 10 WKY rats were placed individually in transparent resin glass cages (31 cm × 23 cm × 16 cm). According to Fabrizio Sanna study,^35^ after a 30‐minute habituation period, rats were treated with APO (dissolved in saline, 0.2 mL/rat) by subcutaneous injection. After treatments, rats were observed for 30 minutes in order to count the times of penile erection and yawning episodes. Penile erections were scored when the penis emerged from the penile sheath, which was usually accompanied by penile grooming and hip flexions. Yawnings were scored when the mouth open at least 1‐3 seconds of duration, which occasionally accompanied by stretching. Both behavioural responses were recorded by an observer who was not aware of the treatments done.

### 2.3 Evaluation of erectile response in the rat

Erectile function of all animals was evaluated. As previously reported,^36^ rats were anaesthetized with pentobarbital 35 mg/kg intraperitoneal injection. Mean arterial pressure (MAP) was continuously monitored via the carotid artery. MAP and intracavernous pressure (ICP) were recorded through pressure transducers connected to a PowerLab 4/30 data acquisition system (ADInstruments), which was connected in turn to a computer for real‐time monitoring of pressure changes. Pressure transducers were calibrated to water before each experiment. The measurement of erectile response elicited by ES of cavernous nerve was performed as previously described.^37^ ES (width 5 milliseconds, duration 30 seconds, 2.5 V) at varying frequencies (1, 2, 4, 8, 16, 32 Hz) was performed with a 5‐minute interval between each stimulation. According to our previous study,^38^ the erectile response elicited by ES was quantified by calculating the maximal ICP/MAP ratio. If the ratio is over 0.6, the erectile function is regarded as normal, which was suggested by Melman et al^39^ at the Albert Einstein College of Medicine in New York. The area under the curve (AUC) of ICP curve was calculated used Image pro plus 6.0 (Media Cybernetics, Inc).

### 2.4 Tissue preparation

After anaesthetization by isoflurane (in 100% oxygen, 5% for induction and 1.5% for maintenance) at a flow rate of 1 L/min, rats were killed by cervical dislocation and their penes were quickly obtained. The skin, urethra, superficial blood vessels and nerves were removed. Tissue from 10 SHR rats and 10 WKY rats were used for histological experiments, RNA extractions and protein extractions. Each tissue was cut into three pieces. One piece was placed into 10% formalin for histological studies and another two pieces were snap‐frozen in liquid nitrogen and stored at −80°C for RNA extraction and protein extraction. Moreover, tissues from another 10 SHR rats and 10 WKY rats were used for detection of ROS level and antioxidant capacity. Thus, each tissue was cut into five pieces for each different assay. Additionally, tissues from 10 SHR and WKY rats were used to determine the in vitro activity of PDE5 enzyme.

### 2.5 Total RNA extraction and real‐time RT‐PCR

Total RNA was extracted from the frozen tissues using TRIzol reagent (Invitrogen) according to the manufacturer's protocol and quantitated by a Nanodrop spectrophotometer (Bio‐Rad). Next, 2 μg of RNA was used to perform reverse transcription using the SuperScript II First‐Strand Synthesis System (Invitrogen) according to the manufacturer's protocol. For each sample, 100 ng of cDNA was used to perform RT‐PCR using a Bio‐Rad CFX96 system based on SYBR green incorporation and fluorescence and each determination was repeated independently three times for analysis. For rat CC tissue, the following targets were amplified: *PDE5A*, *NOS* isoforms and α1‐adrenoreceptor isoforms (*α1aAR*, *α1bAR* and *α1dAR*). Primer sequences are shown in Table 1. For relative quantification, gene expression was normalized to expression of the *β‐actin* housekeeping gene and compared by the 2^−ΔΔCT^ method.

**TABLE 1:** Primer sequences used to amplify target genes by real‐time RT‐PCR

Target gene	Primer sequence
*PDE5A*	
Forward	5′‐TTGGAGAGCCCTTGAACATCA‐3′
Reverse	5′‐GTAGCCTGTAATTTGGTCAACTTCTG‐3′
*α1aAR*	
Forward	5′‐GCCCTTCTCTGCCATCTTG‐3′
Reverse	5′‐GGCCGCCCAGATATTGC‐3′
*α1bAR*	
Forward	5′‐CCAGGAGTTCCATAGCTGTCAAAC‐3′
Reverse	5′‐CCGACTACAATGCCCAAGGT‐3′
*α1dAR*	
Forward	5′‐TGCGCCACTCGCTCAA‐3′
Reverse	5′‐CCAAAGCAGAGCCAGAATGG‐3′
*eNOS*	
Forward	5′‐GCCTGAGCAGCACAAGAGTTAC‐3′
Reverse	5′‐CCAGCCCAAACACACAGAACC‐3′
*nNOS*	
Forward	5′‐GGCAAACATGACTTCCGAGTGT‐3′
Reverse	5′‐CCCCAAGGTAGAGCCATCTG‐3′
*β‐actin*	
Forward	5′‐ACCAACTGGGACGATATGGAGAAGA‐3′
Reverse	5′‐TACGACCAGAGGCATACAGGGACAA‐3′

### 2.6 SDS‐PAGE and western blotting analysis

As previously described,^40^ proteins were extracted from frozen samples using RIPA reagent with freshly added phosphatase and protease inhibitors (Sigma‐Aldrich). For each sample, 20 μg of total protein was electrophoresed on a 10% sodium dodecyl sulphate‐polyacrylamide (SDS‐PAGE) gel (Epizyme Biological Technology Ltd) and transferred to polyvinylidene fluoride (PVDF) membrane (Millipore) using a Bio‐Rad wet transfer system. The membrane was blocked for 2 hours at room temperature in TBST (Tris‐buffered saline with 0.05% Tween 20) containing 5% [w/v] non‐fat dry milk solution and incubated overnight at 4°C with primary antibodies (Table 2). After washing three times with TBST, the membranes were incubated at room temperature for 2 hours with secondary antibody. Detection of reaction antigen was performed with an enhanced chemiluminescence (ECL) kit (Epizyme Biological Technology Ltd). The bands were quantified using a Bio‐Rad Molecular Imager^®^ ChemiDoc™ XRS+ System and Quantity One^®^ 1‐D Analysis software (Bio‐Rad). The expression levels of target protein were normalized to the expression of β‐actin.

**TABLE 2:** List of primary antibodies

Protein target	Name of antibody	Manufacturer and catalog	Species raised in; monoclonal or polyclonal	Dilution used
α‐SMA	Smooth muscle actin antibody (B4)	Santa Cruz, sc‐53142	Mouse monoclonal	1:1000
Collagen I	Anti‐collagen I antibody	Abcam, ab34710	Rabbit polyclonal	1:1000
β‐actin	β‐actin (C4)	Santa Cruz, sc‐47778	Mouse monoclonal	1:1000
SMMHC	MYH11 antibody (G4)	Santa Cruz, sc‐6956	Mouse monoclonal	1:100
PDE5	Anti‐PDE5A/PDE5 antibody	Abcam, ab64179	Rabbit polyclonal	1:1000 (WB) 1:100 (IF)
P‐PDE5	Phospho‐PDE5A antibody	FabGennix, PPD5A‐140AP	Rabbit monoclonal	1:500
eNOS	NOS3 antibody (B‐5)	Santa Cruz, sc‐136977	Mouse monoclonal	1:1000
nNOS	NOS1 antibody (A‐11)	Santa Cruz, sc‐5302	Mouse monoclonal	1:1000
α_1a_AR	α_1a_AR antibody	Santa Cruz, sc‐100291	Mouse monoclonal	1:1000
α_1b_AR	Anti‐ADRA1B antibody	Abcam, ab169523	Rabbit monoclonal	1:1000
α_1d_AR	Anti‐ADRA1D antibody	Abcam, ab3462	Rabbit polyclonal	1:1000
BiP	GRP78/BiP antibody	Abclonal, A0241	Rabbit polyclonal	1:1000
CHOP	DDIT3/CHOP antibody	Abclonal, A6504	Rabbit polyclonal	1:1000
Nox1	NOX1 antibody	Abclonal, A12309	Rabbit polyclonal	1:1000
Nox4	Anti‐NADPH oxidase 4 antibody	Abcam, ab133303	Rabbit monoclonal	1:1000
SOD2	Anti‐SOD2/MnSOD antibody	Abcam, ab13533	Rabbit polyclonal	1:1000
Catalase	Anti‐catalase antibody	Abcam, ab16731	Rabbit polyclonal	1:1000
Bcl‐2	BCL2 antibody	Abclonal, A0208	Rabbit polyclonal	1:1000
BAX	BAX antibody	Abclonal, A0207	Rabbit polyclonal	1:1000

### 2.7 Masson's trichrome staining

As previous described,^40^ after being fixed in 10% neutral buffered formalin for 48 hours, CC tissues were embedded into paraffin and cut into 10 μm sections. Then, the sections were stained by Masson composite staining solution (Fuzhou Maxim Biotech Co., Ltd.) and scanned into electronic micrographs using an Aperio VERSA 8 (Leica CM 1850). The CCSM cells were stained dark red, collagen fibres were stained blue and epithelial cells were stained red. For each sample, we analysed three areas under magnification (×100). The per cent area of SM, collagen fibres and epithelium were quantitated with Image pro plus 6.0 (Media Cybernetics, Inc), respectively. Specifically, we took the whole area (sinusoidal space not included) of the CC as 100%, and then calculated the per cent area of each component.

### 2.8 Immunofluorescence microscopy

Rat CC were embedded in Tissue‐Tec OCT compound (SakuraFinetek Japan) and snap‐frozen. Then, the tissue was sectioned into 10 μm thick slices, thawed and then mounted onto glass slides using a cryostat (Leica CM 1850). After being air‐dried, sections were fixed for 10 minutes in ice‐cold acetone and washed in PBS. Then, sections were incubated for 2 hours in a mixture of PBS supplemented with 0.2% Triton X‐100 and 0.1% bovine serum albumin. After incubation overnight with the primary antibody mixture of SMMHC (smooth muscle myosin heavy chain, mouse polyclonal to MYH11 [Myosin Heavy Chain 11], 1:100) and PDE5 antibody (rabbit polyclonal to PDE5A, 1:100), the secondary antibodies (Jackson ImmunoResearch Inc) labelled with FITC‐conjugated anti‐mouse IgG (1:200) and Cy3‐conjugated anti‐rabbit IgG (1:1000) were used to visualize the localization of the two primary antibodies. DAPI was used for staining the nucleus. Negative controls were performed for all samples by omitting the primary antibodies. Rat lung tissue was used as a positive control for PDE5A staining. Stained sections were viewed by a laser microscope (Olympus). Analysis was performed using the NIS‐Elements Viewer 3.20 (Nikon).

### 2.9 PDE5 activity assay

The CC tissue was immediately frozen in liquid nitrogen and crushed into a powder with grinding rods. Then, the powder was placed into a centrifuge tube containing lysis buffer and vortexed for 30 seconds. After storage at 4°C for 30 minutes, the homogenate was centrifuged for 15 minutes at 12 000 *g*; then, the supernatant was used for protein quantification using the BCA method and lysates containing 5 mg of total protein were used for the following PDE5 activity assay.

Buffer containing Tris (50 mmol/L, pH 7.5), indomethacin (10^−5^ mol/L), nitro‐l‐arginine (10^−4^ mol/L), MgCl_2_ (10^−5^ mol/L), bovine serum albumin (0.3 mg/mL) and cGMP (guanosine 3′, 5′‐cyclic monophosphate sodium salt, 2 × 10^−6^ mol/L) were added into tissue lysates and then incubated at 30°C for 15 minutes. After stopping the reaction via administration of HCl (250 mmol/L), the level of excess cGMP in the reaction solution was determined by specific commercial ELISA kit (ab65356; Abcam). As cGMP was hydrolysed by PDE5, the amount of cGMP detected was negatively correlated with the activity of PDE5.

### 2.10 Superoxide assay

Superoxide levels were measured using a commercially available assay kit (S0060; Beyotime Biotechnology). According to the manufacturer's instruction, CC tissue samples were homogenized in a ratio of 212 μL of detection reagent per 5 mg of tissue. Detection reagent consisted of 200 μL phosphate buffer (composition in mmol/L: NaCl 68.9; Na_2_HPO_4_ 4.08; KH_2_PO_4_ 0.73; KCl 1.34; pH 7.4), 10 μL WST‐1 solution and 2 μL catalase solution. Then, the homogenate was incubated at 37°C for 1 hour. The absorbance was recorded at 450 nm reflecting the superoxide level.

### 2.11 H_2_O_2_ (hydrogen peroxide) assay

The H_2_O_2_ level was determined using a commercially available assay kit (S0038; Beyotime Biotechnology) according to the manufacturer's instructions. Briefly, CC tissue samples were homogenized in a ratio of 100‐200 μL of lysate buffer per 5‐10 mg of tissue. After homogenization, the supernatant was centrifuged for 5 minutes at 12 000 *g* for subsequent H_2_O_2_ determination. Following the indicated treatment, the detection reagent (100 μL/well) was added for 20 minutes at room temperature. The absorbance was recorded at 560 nm reflecting the H_2_O_2_ level.

### 2.12 MDA (malondialdehyde) assay

The MDA level was examined using an MDA assay kits (S0131; Beyotime Biotechnology) according to the manufacturer's instructions. Briefly, CC tissue samples were homogenized in a ratio of 100‐200 μL of lysate buffer per 5‐10 mg of tissue. After homogenization, the supernatant was centrifuged for 5 minutes at 12 000 *g* for subsequent determination. Following the indicated treatment, the supernatant (100 μL/sample) and detection reagent (200 μL/sample) were added to a tube. After mixing, this mixture was heated for 15 minutes at 100°C. Then, the mixture was cooled to room temperature and centrifuged for 10 minutes at 100 *g*. The absorbance of supernatant (200 μL/sample), reflecting the MDA level, was determined at 532 nm.

### 2.13 SOD (superoxide dismutase) activity assay

SOD activity in the rat CC was measured using an assay kit (#19160; Sigma‐Aldrich) according to the manufacturer's instructions. The CC homogenates were prepared in phosphate buffer (composition in mmol/L: NaCl 68.9; Na_2_HPO4 4.08; KH_2_PO4 0.73; KCl 1.34; pH 7.4). SOD activity was measured in the supernatant (20 μL/sample). The absorbance was measured at 450 nm, and SOD activity was expressed as an inhibition rate per cent per milligram protein.

### 2.14 Catalase activity assay

Catalase activity was assayed by H_2_O_2_ consumption. CC tissue was homogenized in 0.2 mL of phosphate buffer (68.9 mmol/L of NaCl, 4.08 mmol/L of Na_2_HPO4, 0.73 mmol/L of KH_2_PO4, and 1.34 mmol/L of KCl [pH 7.4]). One hundred microlitres of phosphate buffer (K_2_HPO4, 0.1 mmol/L, KH_2_PO4, 0.1 mmol/L [pH 6.5]) was added to the homogenates that were then centrifuged at 12 000 *g* for 10 minutes at 4°C. Reaction buffer (2.5 mL of Tris EDTA buffer [1 mol/L Trizma and 5 mmol/L of EDTA], 47.35 mL of MilliQ water, and 175.5 μL of H_2_O_2_ 30%) was used to analyse the samples. Reaction buffer (980 μL) was added to quartz cuvettes containing 20 μL of the supernatant. The absorbance was recorded at 240 nm. One catalase (CAT) unit (U) was defined as the amount of enzyme required to decompose 1 mmol/L of H_2_O_2_/min.

### 2.15 Statistical analysis

Results are expressed as mean ± SEM for n experiments. Statistical analysis used either the Student's *t* test with Excel software (two sample treatments compared). *P* < .05 was considered significant.

## 3 RESULTS

As described in Table 3, the MAP of the SHR rats was significantly higher than that of the WKY rats, which was (180.60 ± 9.83) mm Hg vs (112.64 ± 9.57) mm Hg (*P* < .01). The bodyweight of SHR rats was lower than that of WKY rats, which was (257.8 ± 7.5) g vs (293.5 ± 10.5) g (*P* < .05). The baseline ICP was found to be not significantly different between the two groups.

**TABLE 3:** Bodyweight, MAP and baseline ICP in study rats

	Bodyweight (g)	MAP (mm Hg)	Baseline ICP (mm Hg)
WKY (n = 20)	293.5 ± 10.5	112.64 ± 9.57	9.6 ± 0.47
SHR (n = 20)	257.8 ± 7.5*	180.60 ± 9.83**	9.5 ± 1.04

Note

Data are expressed as mean ± SD.

* *P* < .05 vs WKY.

** *P* < .01 vs WKY.

**FIGURE 1:**
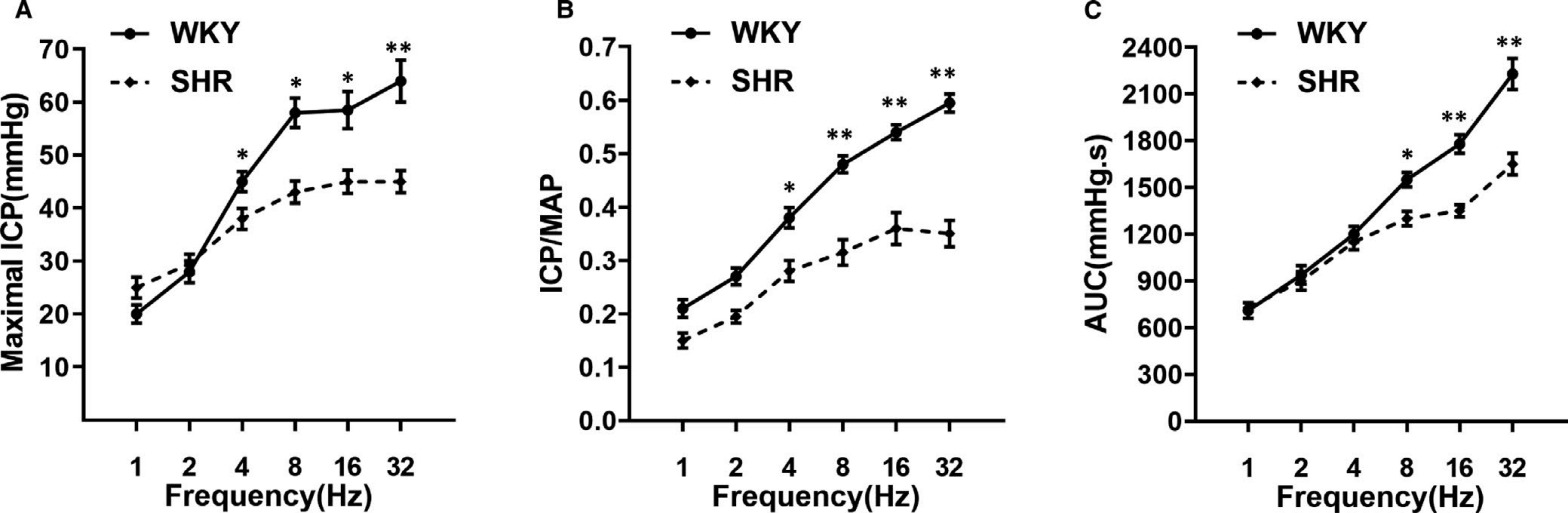
Evaluation of erection function. A, Intracavernous pressure (ICP) measurements from the WKY and SHR in response to electrical stimulation (ES) of the cavernous nerve at various frequencies. B, Maximal ICP normalized by mean arterial pressure (MAP). The maximal ICP is the highest pressure reached in response to stimulation, with MAP being the mean arterial pressure during the plateau phase. C, The area under curve (AUC) of tracings of intracavernous pressure (ICP) curve from WKY and SHR. Data were shown as mean ± SD. *P < .05 vs SHR; **P < .01 vs SHR (n = 20 different rats for each group)

SHR rats were found to exhibit obvious ED, as demonstrated in Table 4, with the numbers of erections induced by APO at (1.6 ± 0.7) vs (4.2 ± 0.2) in WKY rats (*P* < .01). Also, the number of yawns induced by APO was (1.4 ± 0.7) in SHR vs (9.2 ± 1.9) in WKY rats (*P* < .01). In line with the APO testing, both the maximal ICP rise elicited by ES of the cavernosum nerve and ICP normalized to MAP were significantly decreased in SHR rats at the varying stimulation frequencies (Figure 1A,B), when compared with WKY rats. In addition, the AUC of the ICP curve was decreased at the stimulation frequency of 8 Hz (*P* < .05), 16 Hz and 32 Hz (*P* < .01) (Figure 1C).

**TABLE 4:** Times of erection and yawning after apomorphine treatment

	Erection	Yawning
WKY (n = 10)	4.2 ± 0.2	9.2 ± 1.9
SHR (n = 10)	1.6 ± 0.7**	1.4 ± 0.7**

Note

Data are expressed as mean ± SD.

** *P* < .01 vs WKY.

**FIGURE 2:**
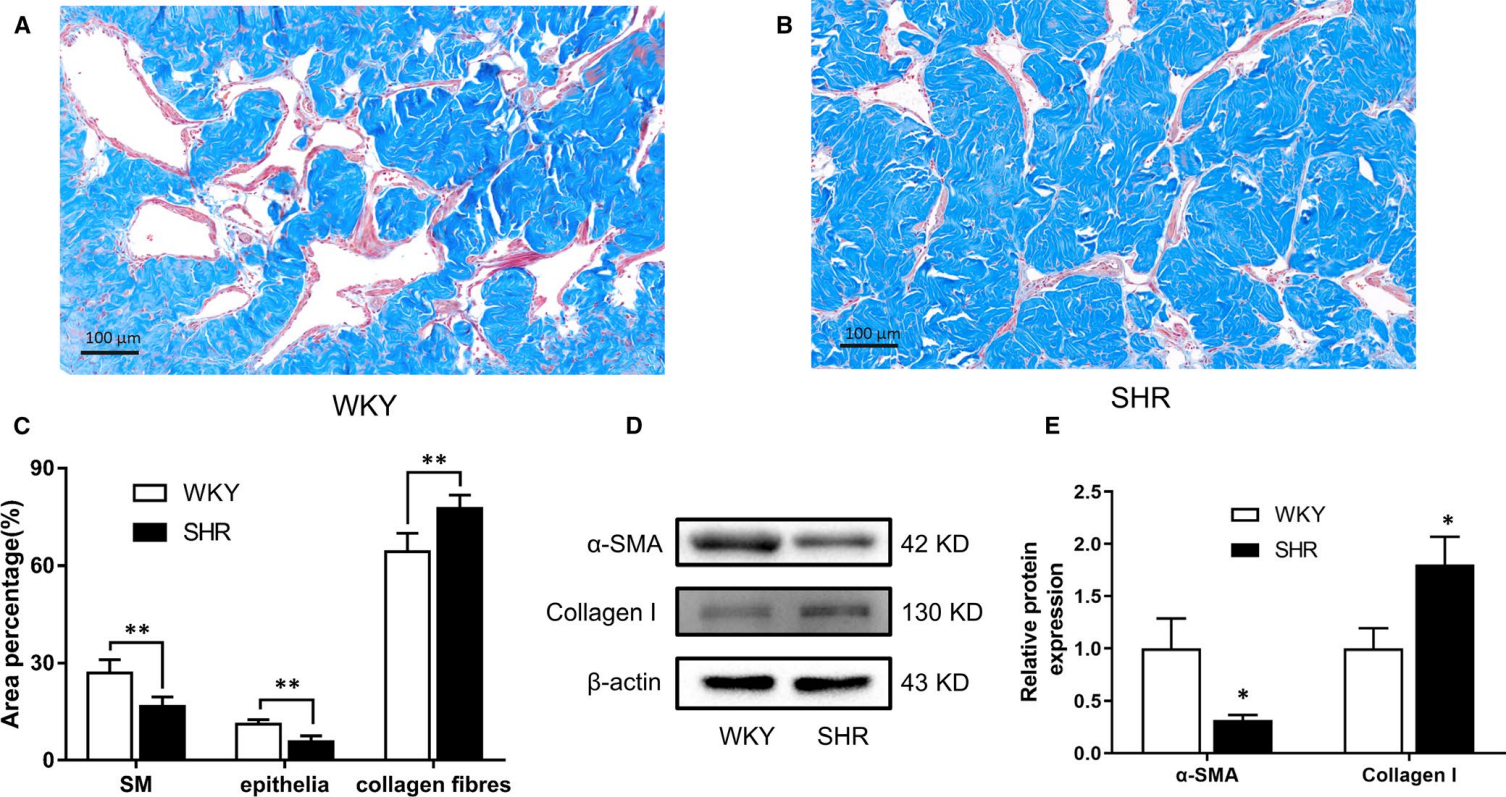
Masson's trichrome staining of rat CC and Western blotting analysis. Masson's trichrome staining for WKY (A) and SHR (B) rat CC, respectively (magnification ×200). CC smooth muscle (SM) cells were stained dark red, collagen fibres were stained blue and epithelial cells were stained red. C, The bar graph for area percentage of each different component (SM, epithelia and collagen fibres) between WKY and SHR (n = 20 different animals for each group). D, Typical bands of α‐SMA and collagen I protein. E, Western blot analysis of protein expression. White column, WKY; black column, SHR. Quantification of protein expression is calculated by the grey value ration of target protein/β‐actin (n = 10 different animals for each group). Data were shown as mean ± SEM. *P < .05 vs WKY; **P < .01 vs WKY

**FIGURE 3:**
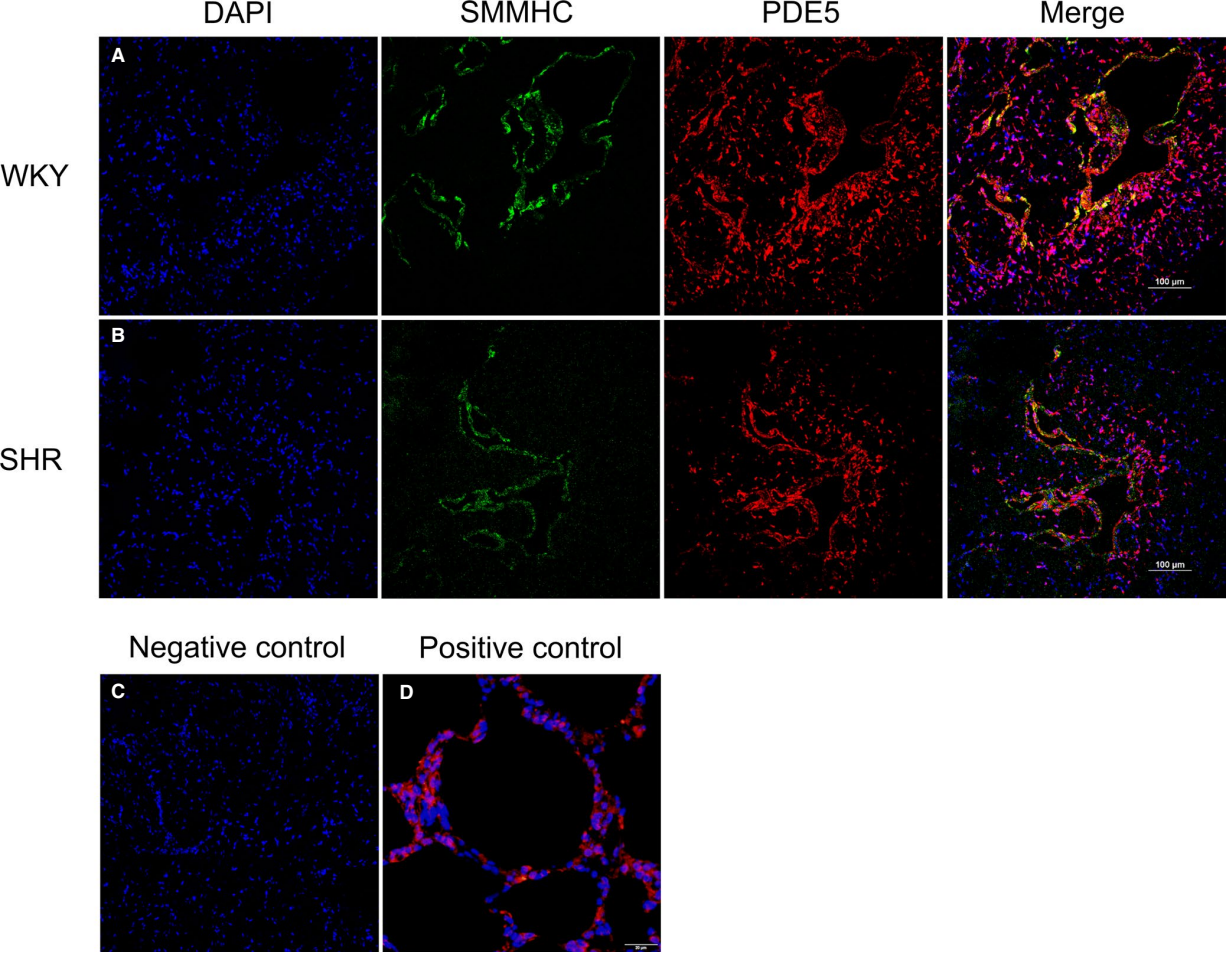
Immunolocalization of PDE5 in rat CC. Representative double immunofluorescence staining for WKY (A) and SHR (B) CC was conducted. DAPI (blue fluorescence), SMMHC (green fluorescence), PDE5 (red fluorescence) and merged images were showed from left to right (magnification ×200). C, Negative control by omitting the primary antibody (magnification ×200). D, Rat lung tissue used as positive control for PDE5 (magnification ×400)

Masson's trichrome staining revealed histological changes in SHR CC. As demonstrated in Figure 2, the percentage of smooth muscle (*P* < .01) and epithelia (*P* < .01) were significantly decreased in the CC of SHR rats, while the percentage of collagen fibres (*P* < .01) were relatively increased. In line with histological results, α‐SMA (smooth muscle marker) protein and collagen I (collagen fibre marker) protein were down‐regulated (*P* < .05) and up‐regulated (*P* < .05), respectively. The localization of PDE5 in rat CC was determined using confocal microscopy. As shown in Figure 3, PDE5 was present both in SM and endothelial cells. Moreover, the staining was less in SHR than that in WKY rats. A negative control was performed by omitting the primary antibody (Figure 3C). Rat lung tissue was used as positive control for PDE5 (Figure 3D).

**FIGURE 4:**
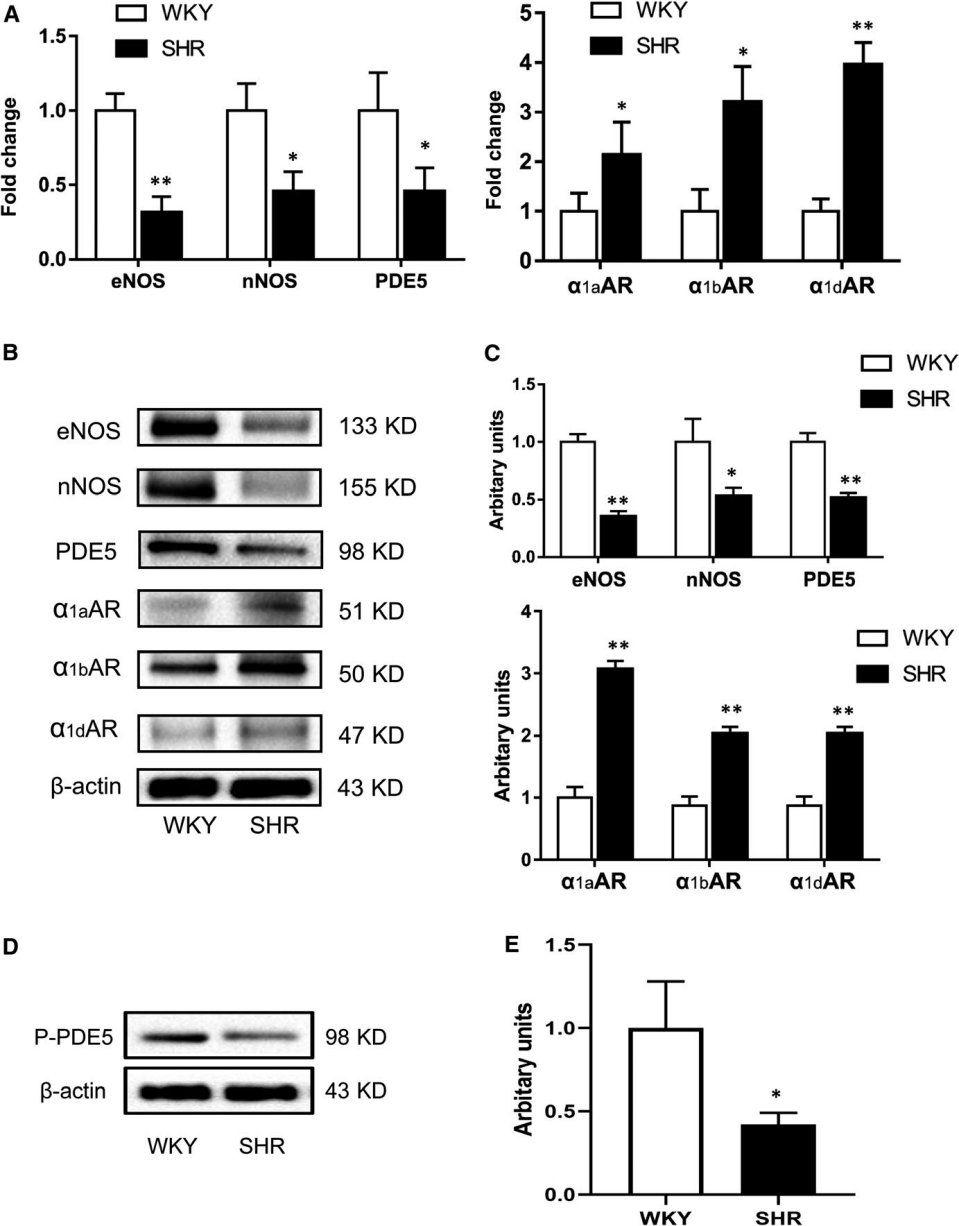
Expression of NOS isoforms, PDE5, phosphorylated PDE5 (Ser‐92) and alpha‐1 adrenergic receptor isoforms in rat CC. A, Relative expression of NOS isoforms, PDE5 and alpha‐1 adrenergic receptor isoforms at mRNA level. White column, WKY; black column, SHR. B, Typical bands of NOS isoforms, PDE5 and alpha‐1 adrenergic receptor isoforms proteins. Molecular weight (KD) is indicated to the right of the blot. C, Western blot analysis of protein expression of NOS isoforms, PDE5 and alpha‐1 adrenergic receptor isoforms. White column, WKY; black column, SHR. Quantification of protein expression is calculated by the grey value ration of target protein/β‐actin. D, Typical bands of P‐PDE5 (Ser‐92) and β‐actin. Molecular weight (KD) is indicated to the right of the blot. E, Western blot analysis of protein expression of P‐PDE5. White column, WKY; black column, SHR. Quantification of protein expression is calculated by the grey value ration of target protein/β‐actin. Data are expressed as mean ± SEM of arbitrary unit. Experiments were repeated three times for each sample (n = 10 for each group). *P < .05 vs WKY; **P < .01 vs WKY

Next, the expression of important molecules in the relaxation and contraction pathways of CCSM was determined. As shown in Figure 4A, the mRNA levels of α_1A_, α_1B_ and α_1D_ were elevated with hypertension by 2.1‐fold (*P* < .05), 3.2‐fold (*P* < .05) and 3.8‐fold (*P* < .01), respectively. In contrast, the mRNA levels of PDE5 (*P* < .05), eNOS (*P* < .01) and nNOS (*P* < .05) were attenuated with higher blood pressure. Consistently, protein levels of α_1A_ (*P* < .01), α_1B_ (*P* < .01) and α_1D_ (*P* < .01) were significantly increased while eNOS (*P* < .01), nNOS (*P* < .05) and PDE5 (*P* < .01) were significantly decreased (Figure 4B,C) in hypertensive animals. Moreover, the protein level of phosphorylated‐PDE5 (Ser‐92, P‐PDE5) was determined and it was observed that this active form of PDE5 was also decreased (*P* < .05) in SHR CC tissue (Figure 4D,E). Correspondingly, the in vitro activity of PDE5 in CC tissue was observed attenuated in hypertensive rats (Table 5), which was 213.47 ± 36.43 vs 143.97 ± 27.74 μmol/mg min (WKY vs SHR, *P* < .01), respectively.

**TABLE 5:** Activity of PDE5 in corpus cavernosum of rats

Group	PDE5 activity (μmol/mg min)
WKY (n = 10)	213.47 ± 36.43
SHR (n = 10)	143.97 ± 27.74**

Note

Data are expressed as mean ± SD.

** *P* < .01 vs WKY.

**FIGURE 5:**
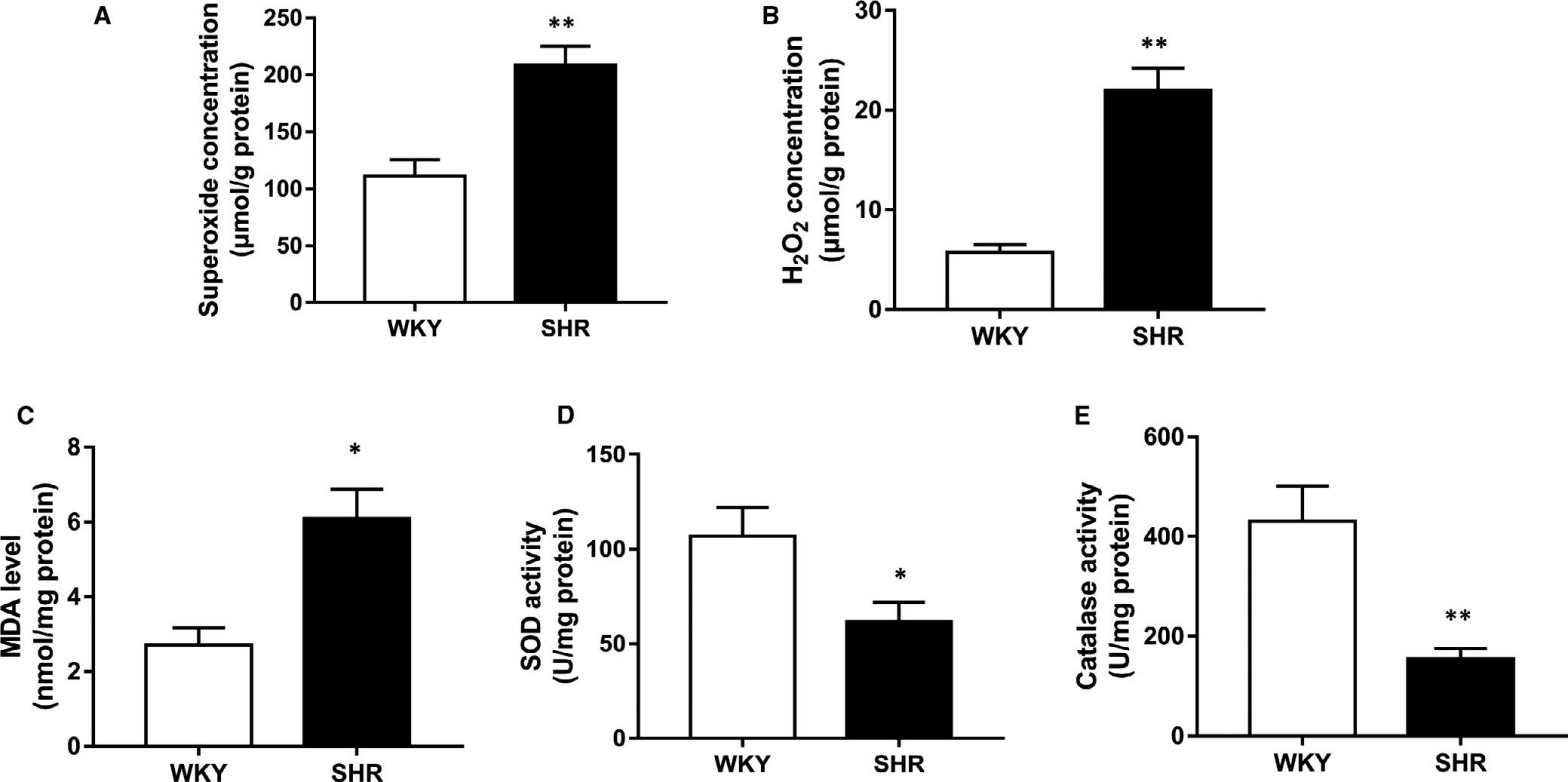
Increased reactive oxygen species (ROS) level and reduced antioxidant level. Oxidative stress and antioxidant level were evaluated by assay kits. Concentration of superoxide (A), H_2_O_2_ (B) showed ROS level in CC tissue. Concentration of MDA (C) showed lipid peroxidation in CC tissue. Activity of SOD (D) and catalase (E) showed antioxidant level in CC. Results are expressed as mean ± SEM for each group (n = 10). *P < .05 vs WKY; **P < .01 vs WKY

Additionally, the ROS level and antioxidant capacity in CC tissue were quantitated. As shown in Figure 5A,B, superoxide (*P* < .01) and hydrogen peroxide (*P* < .01) levels were significantly increased, which suggested excessive ROS levels exist in hypertensive CC tissues. Figure 5C further demonstrated increased MDA (lipid peroxidation marker) in the SHR group (*P* < .05), which indicated oxidative stress. On the other hand, the antioxidant function in hypertensive CC was impaired with the activity of SOD (*P* < .05) and catalase (*P* < .01) attenuated in SHR group (Figure 5D,E).

**FIGURE 6:**
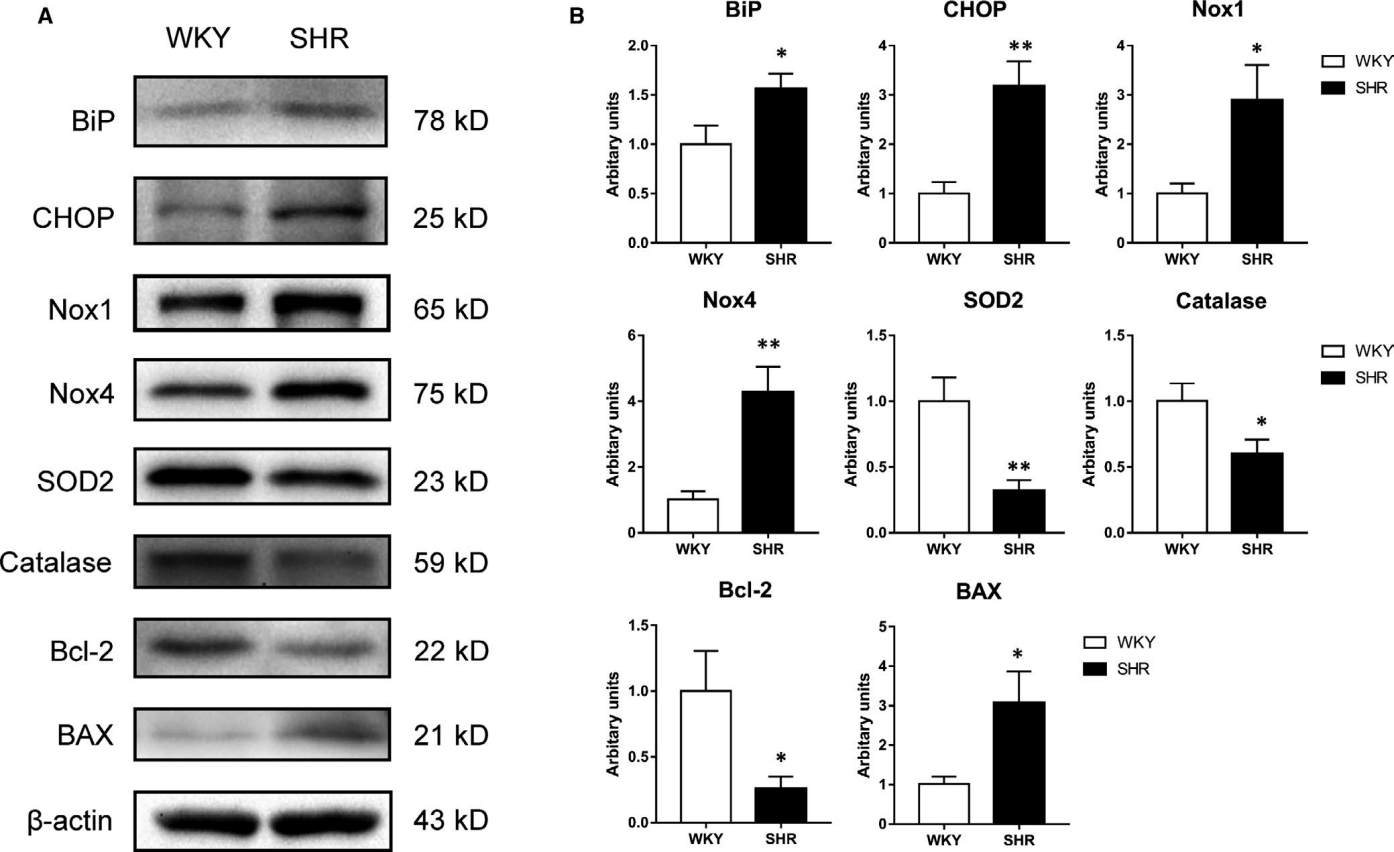
Western blotting results of ER stress, Nox subunits, antioxidant, proliferation and apoptosis in rat CC. A, Typical bands of BiP, CHOP, Nox1, Nox4, SOD2, catalase, Bcl‐2 and BAX proteins. Molecular weight (kD) is indicated to the right of the blot. B, Western blot analysis of protein expression. White column, WKY; black column, SHR. Quantification of protein expression is calculated by the grey value ration of target protein/β‐actin. Data are expressed as mean ± SEM of arbitrary unit. Experiments were repeated three times for each sample (n = 10 for each group). *P < .05 vs WKY; **P < .01 vs WKY

Moreover, the expression of several proteins triggering oxidative stress was examined in the CC of hypertensive rats. As shown in Figure 6A, BiP (glucose‐regulated protein 78/binding immunoglobulin protein) protein and CHOP (C/EBP homologous protein) protein were up‐regulated in SHR group, indicating that the CC of SHR may suffer ER (endoplasmic reticulum stress). Additionally, Nox1 (NADPH oxidase 1) and Nox4 (NADPH oxidase 4) proteins were up‐regulated in the SHR group which may increase ROS production in the CC. In addition, the Bcl‐2 (B cell lymphoma 2) and BAX (bcl‐2‐like protein 4) proteins were down‐regulated and up‐regulated, respectively, which suggests the proliferation level of cells in the CC is decreased and the apoptosis level increased.

## 4 DISCUSSION

The current study demonstrates for the first time that PDE5 expression was down‐regulated and its activity was attenuated in hypertensive rat CC. Our novel data also showed that CC tissue from hypertensive rats exhibited an imbalance between ROS and antioxidants in the penis, which may be associated with the occurrence and development of ED induced by high blood pressure. Our study suggested that hypertensive ED patients may be less responsive to PDE5is and oxidative stress could be used as a new therapeutic target for this type of ED.

Consistent with previous reports that SHR rats often showed impaired erectile function,^41^, ^42^ the present study found hypertension caused a severe ED, as the maximal ICP rise and ICP/MAP ratio to ES, along with the AUC were strongly reduced for hypertensive rats. In line with the decreased ICP induced by ES of the cavernous nerve, both erection and yawn numbers were lessened which were demonstrated by our APO test (Table 4). APO triggers penile erection, and especially yawning, mainly through the central nervous system. Therefore, not only peripheral but also central pathophysiology was associated with hypertensive ED. It is well known that hypertension often leads to arteriosclerosis, including the cerebrovascular system, which might contribute to central ED. However, the exact mechanism needs to be further investigated.

It is well known that NO release and downstream biological actions play the most important role in the process of penile erection and the impairment of NOS may attenuate erectile function.^43^, ^44^ In line with previous studies,^41^, ^42^ the present study per se observed a significant reduction of eNOS and nNOS expression in the CC of SHR rats (Figure 4), which might underlie the impaired erectile response. Long‐term hypertension can produce endothelial injury and function abnormality.^17^ Indeed, our Masson's trichrome staining demonstrated a significant loss of epithelia in SHR CC (Figure 2), which may result in the down‐regulation of eNOS. With regard to the lowered expression of nNOS, previous studies did show that hypertension can damage nerve fibres or neurons in the CC,^19^, ^42^ which could induce the down‐regulation of nNOS. In addition to the decreased relaxation signal, the increased contractile response could further contribute to hypertensive ED. A number of studies have demonstrated that α1‐adrenergic receptors in the CC are modulated by androgens,^45^ ageing^46^ and diabetes mellitus.^47^ It has also been reported that α1‐receptor isoform distribution is changed in the iliac artery of SHR.^48^ Similar to Yono et al,^49^ our study showed that all α1‐adrenergic receptor isoforms were up‐regulated at both the mRNA and protein levels. Therefore, higher expression of α1‐adrenergic receptors may lead to higher contractile responsiveness of CCSM and hence attenuated erection.

Interestingly, our current study found that there was a significantly lower expression of PDE5 in SHR CC both at the mRNA and protein level. Similar to a previous report,^33^ our immunolocalization study observed PDE5 was almost entirely confined to the endothelial cells and smooth muscle cells of blood vessels and cavernous spaces. In recent decades, the modulation of PDE5 was widely studied. The Burnett lab suggested eNOS could modulate PDE5 expression.^50^ They found that in mice with the eNOS gene or both the nNOS and eNOS genes knocked out, the PDE5 expression of the CC decreased and was associated with priapism, while the nNOS gene alone knocked out showed no such manifestations.^50^ Their group further showed 3‐(1,3‐dioxoisoindolin‐2‐yl) benzyl nitrate (compound 4C, a NO donor) can restore PDE5 expression in CC with both the nNOS and eNOS gene knocked out.^51^ Our data also showed that hypertension down‐regulated eNOS in the SHR CC, which may cause the down‐regulation of PDE5. Although the NO‐cGMP‐PDE5 signalling pathway has not been completely proven to have compensation or feedback mechanisms, it is possible that the reduction of NO could be compensated by the decrease of PDE5 to maintain a certain level of cGMP and ultimately maintain partial or satisfactory erectile function, which may explain why not all hypertensive men suffer ED. On the other hand, the down‐regulation of PDE5 could account for less responsiveness to PDE5is for some hypertensive ED patients. Azab et al^31^ demonstrated that PDE5is were less effective in men with hypertension and ED than in ED patients without hypertension. In addition, Goldstein et al^32^ also showed similar results as well as treatment response rates of sildenafil for hypertensive ED patients decreasing with increasing age. As PDE5 was mostly localized in SM, the CCSM loss observed in our present study could also be attributed to the PDE5 reduction. In addition, ischaemia and hypoxia resulting from hypertension may be another mechanism leading to the decrease of PDE5. Previous studies did find decreased blood flow in the SHR penis^49^ and elevated hypoxia marker HIF‐1α.^52^ Another study proved that increased HIF‐1α level could result in PDE5 expression down‐regulated.^53^ However, further investigation is required.

In contrast, two previous studies have demonstrated that PDE5 was up‐regulated in SHR CC tissue. In Shamloul's study,^54^, ^55^ the protein level of PDE5 was observed to increase in SHR, when SD rats were used as a control. However, WKY rats, which were used as a control in our study, were characterized as a genetic control of SHR and used in most studies. Although SD rats and WKY rats are both normotensive, the different genetic traits between two species may effect results. Another study also observed that PDE5 was up‐regulated in SHR using Western blotting and immunohistochemistry.^55^ However, the above two studies only demonstrated the up‐regulation of PDE5 at protein expression level. As PDE5 is an enzyme that hydrolyses cGMP, and its phosphorylation form (P‐PDE5, at Ser‐92) exhibited higher activities, we further determined that the level of P‐PDE5 decreased (Figure 4D,E) and in vitro activity of PDE5 attenuated (Table 5) in SHR, which analyses the alteration of PDE5 in CC tissue of hypertensive rats more comprehensively. Nevertheless, it will be intriguing and more convincing to explore the in vivo functional activity of PDE5 in SHR CC in the future.

It is also noteworthy that CC from SHR exhibited oxidative stress due to an imbalance between excessive ROS levels and impaired antioxidant activity. A previous study has suggested that Nox was a major source of ROS production in penis under the hypertension state.^56^ Our present study found that Nox1 and Nox4 were up‐regulated in the SHR group (Figure 6), accounting for an elevated ROS level in CC. BiP (glucose‐regulated protein 78/binding immunoglobulin protein) is a key regulator of ER stress and its up‐regulation was often used as a marker of ER stress. Our current study found hypertensive animals exhibited ER stress with BiP up‐regulation (Figure 6), which was similar to previous reports.^57^, ^58^ A recent study demonstrated that ER stress was involved in up‐regulation of the Nox protein and subsequent increase of ROS in cultured VSMC from SHR.^28^ On the other hand, down‐regulation of antioxidant enzymes like SOD2 and catalase may exaggerate the accumulation of ROS (Figure 6). Excessive ROS may have some negative effects on erectile function. First, ROS was suggested to damage endothelium, such as scavenging available NO and inducing eNOS uncoupling, which may reduce eNOS protein expression and produce more ROS.^59^ Secondly, ROS may have some histological influence on CC tissue. Our current study observed that the SM component decreased while collagen levels increased in SHR CC (Figure 2), which suggested that CCSM may succumb to apoptosis or fibrosis under high blood pressure. Indeed, CHOP, an apoptosis‐related protein, was up‐regulated and correlated with ER stress (Figure 2). Increased CHOP could lower the anti‐apoptotic protein Bcl‐2 and modify the redox process of the cell, making cells tend towards apoptosis.^60^ We did show that Bcl‐2 decreased but BAX increased in the SHR group (Figure 6). In general, an imbalance between excessive ROS and impaired antioxidant activity leads to persistent oxidative stress, which might play an important role in the pathophysiology of hypertensive ED.

In summary, severe ED was observed in hypertensive animals. Down‐regulation of NOS isoforms and up‐regulation of α1‐adrenergic receptors, along with morphological alterations may be associated with pathophysiology of hypertensive‐related ED. Especially, oxidative stress is associated with the increased contractile responsiveness of tissue from hypertensive rats. Interestingly, PDE5 was found attenuated in CCSM of hypertensive rats. It is possible that the reduction of NO could be compensated by the decrease of PDE5 to maintain a certain level of cGMP and ultimately maintain partial or satisfactory erectile function, which may explain why not all hypertensive men develop ED. On the other hand, the down‐regulation of PDE5 could be contribute to less responsiveness to PDE5is for some hypertensive ED patients.

## CONFLICT OF INTEREST

No potential conflict of interest was reported by the authors.

## AUTHOR CONTRIBUTIONS

**Weixiang He:** Conceptualization (equal); Data curation (equal); Investigation (equal); Methodology (equal); Validation (equal); Visualization (equal); Writing‐original draft (equal); Writing‐review & editing (equal). **Jianmin Liu:** Conceptualization (equal); Data curation (equal); Investigation (equal); Methodology (equal); Validation (equal). **Daoquan Liu:** Conceptualization (equal); Data curation (equal); Investigation (equal); Methodology (equal). **Jundong Hu:** Data curation (equal); Investigation (equal). **Ye Jiang:** Validation (equal). **Mingzhou Li:** Investigation (equal); Validation (equal); Visualization (equal). **Qian Wang:** Investigation (equal); Methodology (equal); Validation (equal). **Ping Chen:** Methodology (equal); Supervision (equal); Validation (equal). **Guang Zeng:** Methodology (equal); Visualization (equal). **Deqiang Xu:** Validation (equal). **Xinghuan Wang:** Project administration (equal); Supervision (equal); Validation (equal). **Michael E. DiSanto:** Project administration (equal); Writing‐review & editing (equal). **Xinhua Zhang:** Conceptualization (equal); Funding acquisition (equal); Project administration (equal); Resources (equal); Supervision (equal); Writing‐review & editing (equal).

## Data Availability

The data used to support the findings of this study are available from the corresponding author upon request.
